# Diagnostic Performance of Glymphatic System Evaluation Using Diffusion Tensor Imaging in Idiopathic Normal Pressure Hydrocephalus and Mimickers

**DOI:** 10.1155/2019/5675014

**Published:** 2019-06-20

**Authors:** Hajime Yokota, Arvind Vijayasarathi, Milos Cekic, Yoko Hirata, Michael Linetsky, Michael Ho, Won Kim, Noriko Salamon

**Affiliations:** ^1^Chiba University, Department of Radiology, Chiba, Japan; ^2^UCLA Department of Radiological Sciences, Los Angeles, CA, USA; ^3^Toho University, Department of Neurosurgery, Ota, Tokyo, Japan; ^4^UCLA Department of Neurology, Los Angeles, CA, USA; ^5^UCLA Department of Neurosurgery, Los Angeles, CA, USA

## Abstract

**Purpose:**

To investigate the pathological change of the glymphatic system in idiopathic normal pressure hydrocephalus (iNPH) using diffusion tensor imaging (DTI) analysis.

**Materials and Methods:**

24 right-handed patients were referred to our hydrocephalus clinic for assessment of ventriculomegaly and gait impairment. 12 of 24 were diagnosed as pseudo-iNPH (piNPH) based on assessment by a neurologist. Diffusivity maps in the direction of the x-axis (right-to-left) (Dx), y-axis (anterior-to-posterior) (Dy), and z-axis (inferior-to-superior) (Dz) were computed. The diffusion map was coregistered to International Consortium for Brain Mapping (ICBM) DTI-81 atlas. The analysis along the perivascular space (ALPS) index was defined as mean (Dxpro, Dypro)/mean (Dypro, Dzasc), where Dxpro and Dxasc are Dx values in the projection and association fiber areas, respectively. Evans index and callosal angle were also assessed on each case.

**Results:**

ALPS indexes of the control, piNPH, and iNPH cases were 1.18 ± 0.08, 1.08 ± 0.03, and 0.94 ± 0.06, respectively, and there were significant differences among the groups (control vs. piNPH, P = 0.003; control vs. iNPH P < 0.001; piNPH vs. iNPH, P < 0.001). Area under curve (AUC) was 0.92, 1.00, and 1.00 on control vs. piNPH, control vs. iNPH, and piNPH vs. iNPH on ROC analysis. Between piNPH and NPH, ALPS index has higher diagnostic performance than Evans index and callosal angle (AUC = 1.00 vs. 0.84, P = 0.028; AUC = 1.00 vs. 0.74, P = 0.016).

**Conclusion:**

Atlas-based ALPS index using the DTI method differentiated among iNPH, piNPH, and controls clearly.

## 1. Introduction

Idiopathic normal pressure hydrocephalus (iNPH) remains challenging to diagnose as both clinical symptoms and imaging findings overlap other diseases. The classical clinical trial is gait dysfunction, cognitive impairment, and urinary incontinence [1]. Gait dysfunction is typically the initial symptom of iNPH and is characterized by magnetic or apraxic gait. However, many diseases can mimic the gait disturbance of iNPH. Patients with iNPH always have ventriculomegaly; however, neurodegenerative disease can atrophy brain tissue and cause secondary ventriculomegaly. Evans index (EI) and callosal angle (CA) are imaging markers used to identify ventriculomegaly related to iNPH; however, their diagnostic performance remains controversial. Proposed clinical criteria require excluding other diseases including neurodegenerative diseases [2], which are difficult entities to definitively diagnose themselves. An objective and effective imaging index for diagnosing and following iNPH would be a valuable tool to better identify and treat patients with this challenging entity.

The glymphatic system depends on convective fluid transport through the perivascular space and interstitial space and has a role in clearing brain metabolites. Cerebrospinal fluid flows into the brain parenchyma through the periarterial space and is transported to the interstitial space via aquaporin-P4 water channels on astrocytic end-feet, which compose the blood-brain barrier. Interstitial fluid flows out through the perivenous space and reaches the cervical lymphatic system [3–5]. Failure of the glymphatic system causes storage of cellular waste products such as amyloid-*β* and tau and is strongly related to the pathophysiology of Alzheimer's disease. Recently, glymphatic MRI using intrathecal contrast agent administration revealed delayed glymphatic clearance and transependymal migration of contrast agents in iNPH [6]. However, this method is invasive, and multiple MRI acquisitions are required before and after intrathecal contrast agent injection.

Taoka et al. proposed a new method to evaluate the glymphatic system using diffusion tensor imaging (DTI) [7]. The authors evaluated water diffusivity along the right-to-left direction of the periventricular white matter, which matched the running direction of the parenchymal vessels of the deep white matter. Expected water diffusivity along this direction could partially reflect the function of the glymphatic system. Using this method, they showed a significant correlation between water diffusivity along the right-to-left direction and severity of cognitive dysfunction in Alzheimer's disease. They also proposed the new quantitative value reflecting glymphatic system activity, DTI analysis along the perivascular space (ALPS) index.

Our purpose was to investigate the pathological change of the glymphatic system in the setting of iNPH using this new method of DTI analysis. Analysis was performed for patients with iNPH, normal controls, and cases who had ventriculomegaly but did not satisfy the iNPH diagnostic requirement. Using these populations, we evaluated the diagnostic performance of ALPS index between iNPH and mimickers.

## 2. Materials and Methods

This study was evaluated and approved by our Institutional Review Board.

### 2.1. Subjects

**Figure 1:**
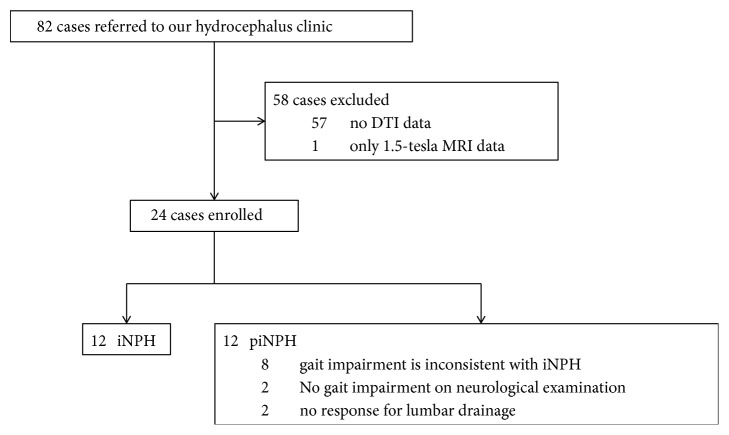
Flow chart of patient inclusion.

The flow chart of patient inclusion is demonstrated in Figure 1. 82 patients were referred to our hydrocephalus clinic from September 2016 to October 2017 for assessment of ventriculomegaly and gait impairment with or without cognitive dysfunction and/or urinary incontinence by a board-certified neurologist with 5 years of experience in adult neurology. We excluded 57 patients without DTI data, and one patient underwent 1.5-tesla MRI. Ultimately, 24 right-handed patients (mean age ± standard deviation, 77.7 ± 8.7 years; 12 men, 12 women) were included in this study. 12 of 24 patients were diagnosed as not having iNPH based on neurological assessment (10 of 12 had gait impairment inconsistent with classic iNPH, and 2 of 12 demonstrated no response after lumbar drainage trial). In the context of this study, this group of patients was included in a group labeled as pseudo-iNPH (piNPH). We also enrolled 12 age-matched control subjects.

### 2.2. MRI Protocol

All MRI were obtained using 3-tesla Siemens Skyra or Prisma MRI scanner. The protocols are listed in Table 1.

### 2.3. Analysis along the Perivascular Space (ALPS) Index

**Figure 2:**
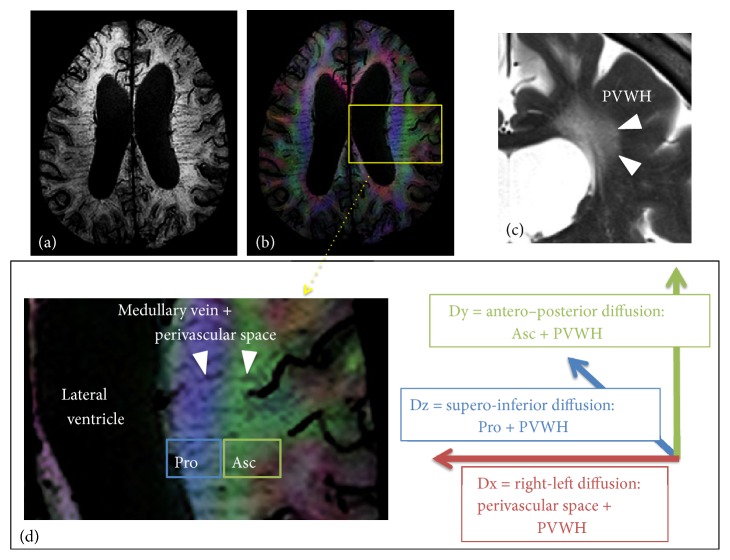
(a) Susceptibility-weighted image (SWI) shows the medullary veins running in the periventricular white matter. (b) Fusion of SWI and color-coded FA map. The periventricular white matter is divided into two regions; medial blue and lateral green areas, which correspond to the projection and association fibers, respectively. (c) In cases with ventriculomegaly, PVWH is often observed on T2-weighted image. (d) The medullary vein runs through the association and projection fiber areas (Pro and Asc). We hypothesized Dx reflects perivascular space of the deep white matter vessels and PVWH. Therefore, Dx partially include the glymphatic system. Dy and Dx are supposed to reflect the association fiber plus PVWH and the projection fiber plus PVWH, respectively.

The schema shows directions of water diffusivity in the periventricular white matter (Figure 2). DTI data was handled by FSL ver. 5.0.9 (https://fsl.fmrib.ox.ac.uk/). Diffusivity maps in the direction of the x-axis (= right-left) (Dx), y-axis (= anterior -posterior) (Dy), and z-axis (= inferior-superior) (Dz) were computed in addition to a fractional anisotropy (FA), mean diffusivity (MD), axial diffusivity (AD), and radial diffusivity (RD) maps. Dx reflects the water diffusivity along the x-axis, which matches the direction of the deep white matter vessels in the periventricular white matter. The glymphatic system exists along the deep white matter vessels, so that Dx should include water diffusivity along the glymphatic system partially. In the periventricular white matter, the projection fibers pass along the wall of the lateral ventricle. In the more lateral area, the association fibers consist of the superior longitudinal fascicles. These fibers pass in the directions perpendicular to the x-axis. We focused on Dx in the projection fiber (Dxpro) and association fiber areas (Dxasc).

Dx is also affected by transependymal edema especially in iNPH. Ischemic white matter change is also common in iNPH [8]. They are represented as periventricular white matter hyperintensity (PVWH). PVWH can increase water diffusivity to all the directions including Dy in the projection fiber area (Dypro) and Dz in the association fiber area (Dzasc), where water diffusivity is originally exiguous. Thus, Dxpro plus Dxasc divided by Dypro plus Dzasc should perform standardization to remove the effect of PVWH. Taoka et al. defined this value as DTI analysis along the perivascular space (ALPS) index:

(1) $$\begin{array}{c} ALPS\;\;index=\frac{mean\left(Dxpro,Dxasc\right)}{mean\left(Dypro,Dzasc\right)} \end{array}$$

Mean (Dxpro, Dxasc) and mean (Dypro, Dzasc) were expressed as Dxmean and Dyzmean in the following session.

### 2.4. Measurement Method of ALPS Index

**Figure 3:**
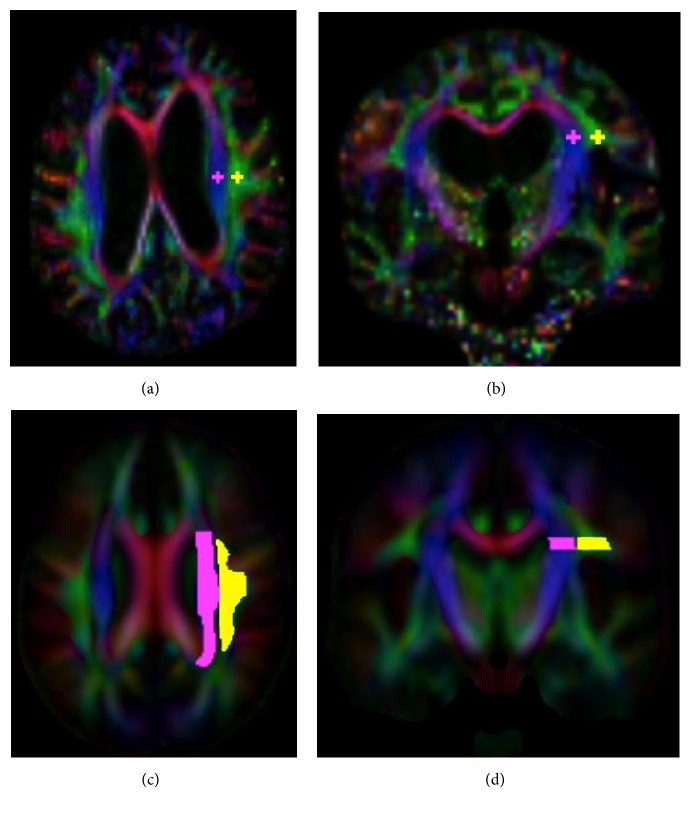
Region of interest (ROI) setting on manual (a, b) and atlas-based (c, d) ALPS indexes. On the manual method, 5-mm-diameter spheres were placed on the projection and association fiber areas with reference to a color-coded FA map on each patient. On the atlas-based method, the labels of the projection and association fiber areas were defined by the labels of the ICBM DTI-81 Atlas. Values of coregistered maps were retrieved using these labels.

Similar to Taoka et al., we manually set 2 regions of interest (ROI) of a 5-mm-diameter sphere in the areas of the left projection and association fibers, respectively (Figures 3(a) and 3(b)). All cases were right-handedness. Thus, the left periventricular white matter was used to compute ALPS index because the association fiber is thicker in the dominant hemisphere. The locations were confirmed by using a color-coded FA map on each patient. A slice showing the brightest color of the projection and association fibers was selected and set ROIs in the centers of these fibers.

An FA map of each case was coregistered to the FA map template of ICBM DTI-81 Atlas by using ANTs (http://stnava.github.io/ANTs/). The accuracy of coregistration was confirmed visually. The other DTI maps were warped using the registration matrix of FA map too. ICBM DTI-81 Atlas has the labels of the projection (superior and posterior corona radiata) and association (superior longitudinal fasciculus) fibers in the periventricular area. We extracted the limited range of the left projection and association fibers, where the x-axis line corresponded to the passing direction of the vessels in the deep white matter (Figures 3(c) and 3(d)). ALPS index was then computed automatically in these labels.

### 2.5. Conventional Measures

EI and CA were evaluated by two raters (A and B with 12 and 14 years of experience for neuroradiology) blinded to final diagnosis according to the commonly used methods on three-dimensional T1-weighted image [9]. EI was defined as the ratio between the largest width between the right-left frontal horns of the lateral ventricles and the largest width of the skull at a reconstructed axial slice. CA was defined as the angle between the superior walls of the right and left lateral ventricles. It was measured at a coronal slice passing through the posterior commissure and perpendicular to the anterior-posterior commissure line. PVWH was classified into four grades in a coronal slice of the body of the lateral ventricle level according to the classification by Shinohara et al. [10]: grade 0, no or only spotty; grade 1, extended along the whole periventricular area; grade 2, irregular hyperintensity extending into the deep white matter; grade 3, extending throughout deep and subcortical white matter. PVWH was evaluated only in the left periventricular white matter where we computed ALPS index. The volume of the supratentorial ventricles (lateral and third ventricles) was manually segmented on two cases of control and iNPH groups. Each ventricular segment was coregistered to the other control or iNPH/piNPH cases using ANTs and was manually modified.

### 2.6. Statistical Analysis

All statistical analyses were performed with R version 3.3.3 (The R Foundation for Statistical Computing, Vienna, Austria). Shapiro-Wilk test was used to check normality. The iNPH and piNPH groups were age-matched by coincidence. Thus, we performed paired tests with Bonferroni correction if applicable. Evaluating patient characteristics, paired t-test, Wilcoxon rank sum test, and Fisher's exact test were used for continuous grading and categorical values, respectively. To evaluate the accuracy of coregistration to ICBM DTI-81 Atlas, FA, MD, AD, and RD of the left projection fiber area were calculated. These values have been assessed in several prior studies [11, 12]. Manual and atlas-based ALPS index were compared with box-plot and paired t-test. Dxmean and Dyzmean derived from atlas-based method were also compared. Using receiver operating characteristic (ROC) curve analysis, diagnostic performances of ALPS index, Evans index, callosal angle, and ventricular volume were evaluated between the control vs. piNPH, control vs. iNPH and piNPH vs. iNPH groups. Area under the curve (AUC) was compared with Delong test. The correlation between atlas-based ALPS index and ventricular volume were assessed using Pearson correlation coefficients. Interobserver reliability of Evans index, callosal angle and manual ALPS index was evaluated using intraclass correlation coefficient (ICC) with a two-way random model. A p value less than 0.05 was considered significant.

## 3. Results

The features of the control, piNPH, and iNPH groups were summarized in Table 2. EIs of all piNPH and iNPH cases were over 0.3. Comparing piNPH with iNPH, there were significant difference in CA and ventricular volume (P = 0.047 and 0.032). By contrast, EI and PVWH grade were not significantly different (P = 0.444 and 0.916). Coregistration of the FA map was visually succeeded in all cases. FA, MD, AD, and RD of the left projection fiber area were significantly increased in iNPH compared with the control group (Table 3). In contrast, there was no significant difference between the piNPH and iNPH groups.

**Figure 4:**
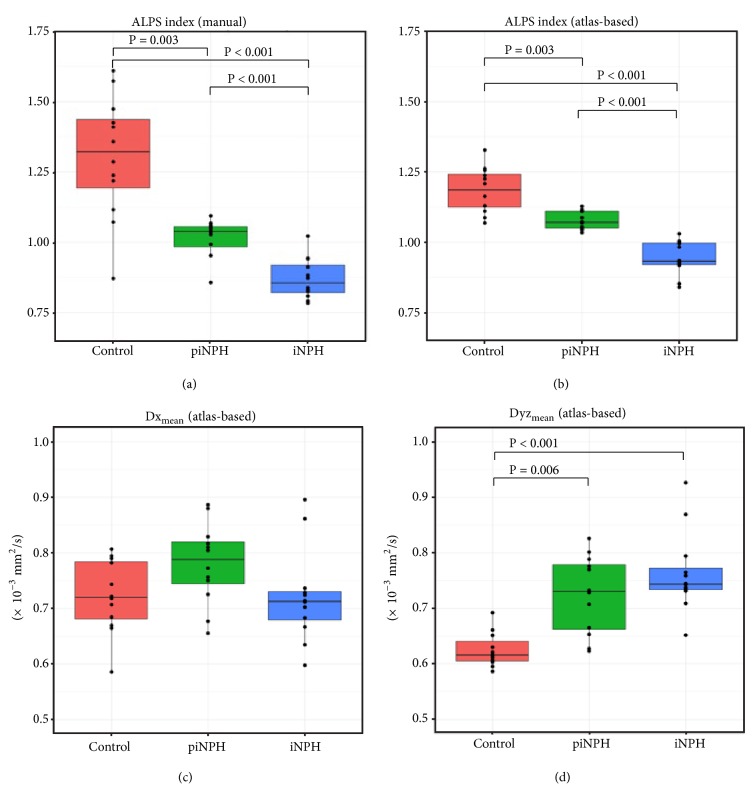
Box-plots of ALPS indexes with the manual (a) and with the atlas-based (b) methods and Dx_mean_ (c) and Dyz_mean_ (d). Only significant P values were shown in the figures. All the values were described in Table 4.

**Figure 5:**
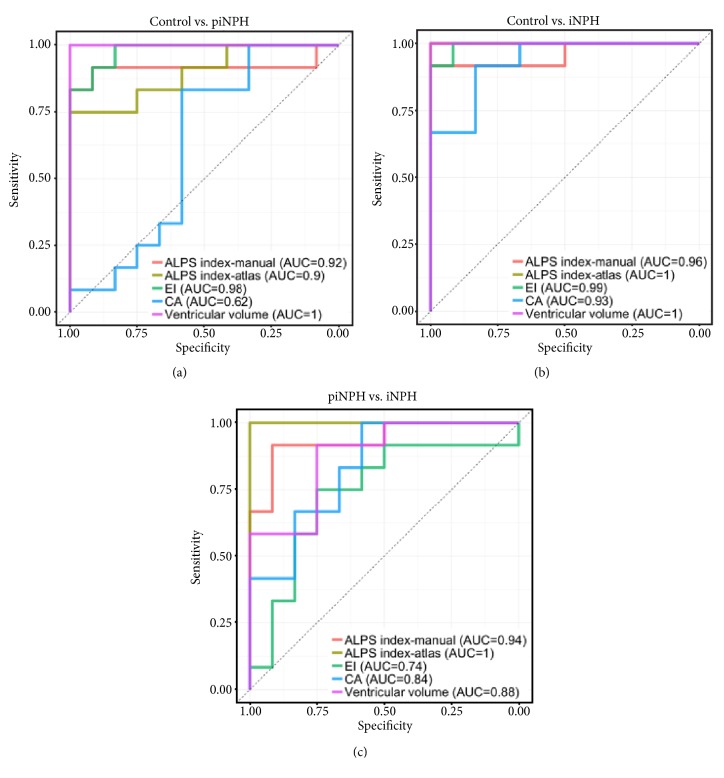
ROC curve analysis between the control vs. piNPH (a), the control vs. iNPH (b), and the piNPH vs. iNPH (c). ALPS index with the manual and the atlas-based methods, EI, CA and supratentorial ventricular volume were assessed.

**Figure 6:**
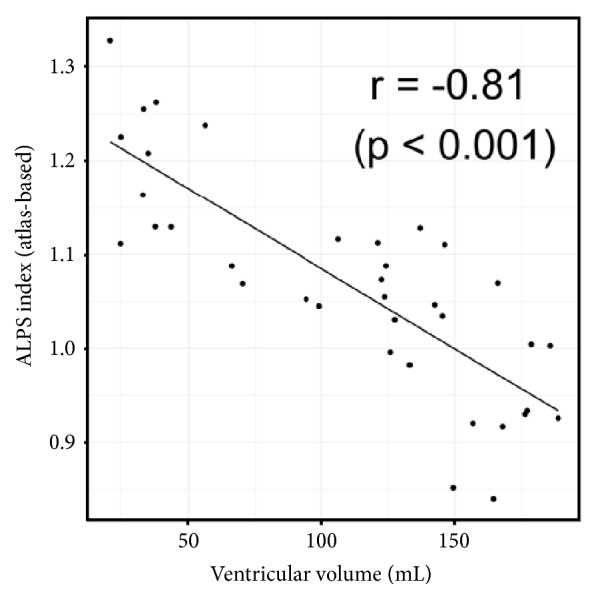
Scatter plot shows correlation between atlas-based ALPS index and supratentorial ventricular volume. There was strong correlation between them (r = -0.81, p < 0.001).

Table 4 and Figure 4 show the results of ALPS index, Dx_mean_, and Dyz_mean_. On manual and atlas-based ALPS indexes, the control group was the highest and followed by piNPH and iNPH groups. Atlas-based ALPS index showed smaller standard deviation compared with manual ALPS index. Thereby, we used atlas-based ALPS index in the following analyses. There were no significant differences of Dx_mean_ among three groups. By contrast, Dyz_mean_ of the control group was significantly higher than the piNPH and iNPH groups (P = 0.001 and 0.006). On ROC curve analysis (Figure 5), atlas-based ALPS index differentiated iNPH from piNPH completely (AUC = 1). The AUCs of atlas-based ALPS index was significantly higher than EI and CA (AUC = 0.74, P = 0.0164; AUC = 0.84, P = 0.048). Also, the AUC was higher than that of ventricular volume; however, there was no statistically significant difference (AUC = 0.88, P = 0.071). Atlas-based ALPS index strongly correlated with ventricular volume (r = -0.81, P < -0.001) (Figure 6). The ICCs of EI, CA, and manual ALPS were 0.85 (P < 0.001), 0.71 (P < 0.001), and 0.81 (P < 0.001), respectively.

## 4. Discussion

Our investigation demonstrates glymphatic system dysfunction in iNPH using the DTI method. The degree of glymphatic dysfunction was expressed as the ALPS index. This index clearly differentiates iNPH, piNPH, and control groups and is related to the degree of ventriculomegaly. ALPS index assessment may have the potential to evaluate the pathophysiological change of iNPH and support a specific diagnosis.

The glymphatic system plays a role in clearing soluble proteins and metabolites from the central nervous system. Astrocytic end-feet formed the fluid transport route through the interstitial space mediated by aquaporin-P4 water channels. The glymphatic system dysfunction leads to deposition of waste including amyloid *β*, resulting in neurodegenerative disease such as Alzheimer's disease. Recently, Ringstad et al. revealed glymphatic system decline in iNPH by using an intrathecal gadolinium contrast agent. In that study, multiple MRI examinations were obtained before and at multiple time points after intrathecal contrast agent administration. However, this intrathecal method requires a lumbar puncture and gadolinium-based contrast, which could result in deposition of gadolinium within the globus pallidus and dentate nucleus [13]. As such, a noninvasive method to assess the glymphatic system is preferable.

Manual and atlas-based ALPS indexes were calculated in this study, and the latter showed stable values with low variation. The manual method is the same as the method used by Taoka et al., who used 5-mm-diameter spheres to measure ALPS index. Our atlas-based method used a wider ROI, which might reflect glymphatic system dysfunction from a larger area. Auto-co-registration is difficult in iNPH patients with severe morphological change, but our method achieved accurate coregistration. FA, MD, AD, and RD were increased in the periventricular projection fiber area in iNPH patients compared to the control group. Moreover, P value of RD was higher than that of AD (P = 0.049 vs. < 0.001). This result matched the findings of previous papers [11, 12]. Interestingly, the piNPH group showed the same tendency as the iNPH group. As such, these values were not useful for differentiation between the piNPH and iNPH groups.

There were no differences of Dx_mean_ among three groups. By contrast, Dyz_mean_ of the piNPH or iPNH groups were significantly higher than the control group. We hypothesized Dx_mean_ reflected water diffusivity along perivascular space and PVWH, and Dyz_mean_ mainly reflected PVWH (Figure 2). Glymphatic system dysfunction might decrease Dx_mean_, whereas PVWH might increase it. The grades of PVWH were similar between piNPH and iNPH (Table 2). Our results of Dx_mean_ and Dyz_mean_ seemed to enhance our hypothesis and implied there was large difference of glymphatic system dysfunction between piNPH and iNPH. ALPS index was computed using the ratio between Dx_mean_ and Dyz_mean_ Therefore, the effect from PVWH might be mitigated by division calculation.

ALPS index correlated with ventricular volume strongly. Pathogenesis of iNPH is still unclear. iNPH presents restricted arterial pulsation secondary to reduced intracranial compliance [13, 14]. Arterial pulsatility is considered to drive water flow of the perivascular space in the mice experiment [15]. Therefore, iNPH should lead to glymphatic system dysfunction caused by water diffusion failure of the perivascular space. Restricted arterial pulsatility and following capillary pulsatility are increasing, resulting in reduced capillary absorption of cerebrospinal fluid and probably leading to ventriculomegaly [14]. Moreover, ventricular reflux is observed in iNPH; the transependymal pressure against the ventricular wall may cause ventriculomegaly [6, 16]. Our results were consistent with previous studies, which demonstrate that glymphatic system dysfunction and ventriculomegaly share pathophysiology.

Diagnosing iNPH is not straightforward. Gait disturbance is typically the initial and most common manifestation, while cognitive impairment and urinary incontinence often follow. Magnetic or apraxic gait, in which the feet appear to be stuck to the walking surface, is a typical finding of gait disturbance. The gait may mimic Parkinsonism with short shuffling steps and stooped, forward-leaning posture. Imaging is key to accurately diagnose iNPH. Ventriculomegaly, prominent sylvian fissures and relative narrowing of the high convexity are representative imaging findings. Although a patient with gait disturbance and ventriculomegaly should be considered to possibly have iNPH, they cannot be considered diagnostic for iNPH as many other neurodegenerative processes have overlapping clinical and imaging findings. To delineate a cutoff of ventriculomegaly, EI and CA have been widely used, as they are easy to measure and reproducible. However, the diagnostic performance of EI and CA is controversial given the overlapping imaging findings seen in other neurodegenerative diseases. Interobserver agreement among neuroradiologists has been modest for differentiating iNPH from Alzheimer's disease or healthy control using visual evaluation with MRI, as accuracy is only 68% - 78% [17]. Additionally, iNPH can share a pathogenic mechanism of Alzheimer's disease presumably due to glymphatic system dysfunction [18]. In the previous studies, iNPH has been compared with healthy controls or Alzheimer's disease. However, in the clinical setting, neurologists aim to differentiate iNPH from not only Alzheimer's disease but also other mimickers which present with ventriculomegaly and symptoms like the classic triad of iNPH. These mimickers can include many types of neurodegenerative disease and may include the very early stage of iNPH. Differentiating iNPH from mimickers via the use of specialized imaging would be very helpful in the appropriate clinical setting. Our results suggest that ALPS index has a potential to differentiate iNPH and mimickers clearly and to better assess the pathophysiological change of iNPH.

There were several limitations in this study. Specifically, the study was retrospective in nature, performed at a single site, and involved a small cohort. Although ALPS index showed adequate diagnostic performance even in our small cohort, a further study with a larger study group is needed to evaluate glymphatic system dysfunction by other factors such as aging. Additionally, the MRI protocols included in this study were mixed. Taoka et al. concluded b factor 1000 was superior to b 2000 to evaluate Alzheimer's disease using ALPS index. Further analysis might be needed to assess a bias from DTI protocols.

## 5. Conclusion

In conclusion, ALPS index using the DTI has a potential to differentiate iNPH from mimickers. The atlas-based method was easy to obtain and provided stable values of ALPS index. ALPS index was associated with ventriculomegaly: therefore, this index might reflect the pathophysiological change of iNPH.

## Figures and Tables

**Table 1:** Imaging protocols.

	Sequence	TR/TE [msec]	Matrix	Resolution [mm]	b-value	MPG [direction]
DTI	EPI/axial	9500-10900/90-100	128x128	2x2x2	0,1200	12
	EPI/axial	7900-12000/66-100	128x128	2x2x2	0,1000	64
	EPI/axial	7600/66	128x128	2x2x2	0,1000	128

		TR/TE/TI [msec]	Flip angle [°]	Matrix	Resolution [mm]	

3D-T1-weighted image	MPRAGE/sagittal	1810-2200/2.23-2.48/900-1100	8-15	256x256x176-192	0.9-1x0.9-1x0.9-1	

		TR/TE [msec]	Matrix	Resolution [mm]		

T2-weighted image	TSE/coronal	3000-9690/98-120	278-345x384-512	0.4x0.4x3-6		

TR/TE/TI = repetition time/echo time/inversion time, MPG = motion-probing gradient.

**Table 2:** Patient characteristics.

	Control	piNPH	iNPH	P values		
	(n = 12)	(n = 12)	(n = 12)	Control vs. piNPH	Control vs. iNPH	piNPH vs. iNPH
Age [years]	75.7 ± 8.4	75.7 ± 9.4	75.3 ± 7.3	1	1	1
Sex [Female, Male]	6, 6	8, 4	4, 8	1	1	1
Clinical symptoms						
Gait disturbance [n]	0	10	12	< 0.001	< 0.001	1
Cognitive impairment [n]	0	7	11	0.014	< 0.001	0.465
Urinary incontinence [n]	0	7	10	0.014	< 0.001	1
Imaging indexes						
Evans index [degree]	26.9 ± 4.1	34.8 ± 3.4	37.2 ± 2.9	< 0.001	< 0.001	0.444
Callosal angle [degree]	126.8 ± 31.2	120.3 ± 35.5	87.7 ± 17.3	1	0.008	0.047
Ventricular volume [mL]	40.3 ± 16.1	127.5 ± 21.1	161.0 ± 22.4	< 0.001	< 0.001	0.032
PVWH grade [n]						
grade 3	0	1	0	0.071	0.314	0.916
grade 2	0	3	2			
grade 1	3	5	5			
grade 0	9	3	5			

Note. Data are shown as mean ± standard deviation.

**Table 3:** DTI changes in periventricular projection fiber area.

	Control	piNPH	iNPH	P values		
	(n = 12)	(n = 12)	(n = 12)	Control vs. piNPH	Control vs. iNPH	piNPH vs. iNPH
FA	0.39 ± 0.03	0.43 ± 0.04	0.46 ± 0.04	0.185	0.013	0.192
MD [× 10^−3^mm^2^/s]	0.77 ± 0.06	0.91 ± 0.08	0.91 ± 0.07	0.002	< 0.001	1
AD [× 10^−3^mm^2^/s]	1.10 ± 0.07	1.34 ± 0.09	1.37 ± 0.08	< 0.001	< 0.001	1
RD [× 10^−3^mm^2^/s]	0.60 ± 0.06	0.69 ± 0.08	0.68 ± 0.08	0.062	0.049	1

Note. Data are shown as mean ± standard deviation.

**Table 4:** ALPS index, Dx_mean_ and Dyz_mean_ of the control, piNPH, and iNPH groups.

	Control	piNPH	iNPH	P values		
	(n = 12)	(n = 12)	(n = 12)	Control vs. piNPH	Control vs. iNPH	piNPH vs. iNPH
ALPS (manual)	1.30 ± 0.22	1.01 ± 0.07	0.87 ± 0.07	0.003	< 0.001	< 0.001
ALPS (atlas-based)	1.18 ± 0.08	1.08 ± 0.03	0.94 ± 0.06	0.003	< 0.001	< 0.001
Dx_mean_ (atlas-based) [× 10^−3^mm^2^/s]	0.72 ± 0.07	0.78 ± 0.07	0.72 ± 0.08	0.230	1	0.360
Dyz_mean_ (atlas-based) [× 10^−3^mm^2^/s]	0.61 ± 0.05	0.72 ± 0.07	0.76 ± 0.07	0.006	< 0.001	0.783

Note. Data are shown as mean ± standard deviation.

## Data Availability

The data used to support the findings of this study are available from the corresponding author upon request.

## Abbreviations

AD: Axial diffusivity
ALPS: Analysis along the perivascular space
CA: Callosal angle
DTI: Diffusion tensor imaging
Dx: Diffusivity along x-axis
Dx_mean_: Mean diffusivity along x-axis in the projection and association fiber areas
Dy: Diffusivity along y-axis
Dyz_mean_: Mean diffusivity along y-axis in the projection fiber area and z-axis in the association fiber area
Pro: Projection
Asc: Association
Dz: Diffusivity along z-axis
EI: Evans index
FA: Fractional anisotropy
iNPH: Idiopathic normal pressure hydrocephalus
MD: Mean diffusivity
piNPH: Pseudoidiopathic normal pressure hydrocephalus
RD: Radial diffusivity.

## References

[1] Adams R. D., Fisher C. M., Hakim S., Ojemann R. G., Sweet W. H. (1965). Symptomatic occult hydrocephalus with normal cerebrospinal-fluid pressure—A treatable syndrome. *The New England Journal of Medicine*.

[2] Relkin N., Marmarou A., Klinge P., Bergsneider M., Black P. M. (2005). Diagnosing idiopathic normal-pressure hydrocephalus. *Neurosurgery*.

[3] Iliff J. J., Wang M., Liao Y. (2012). A paravascular pathway facilitates CSF flow through the brain parenchyma and the clearance of interstitial solutes, including amyloid *β*. *Science Translational Medicine*.

[4] Murtha L. A., Yang Q., Parsons M. W. (2014). Cerebrospinal fluid is drained primarily via the spinal canal and olfactory route in young and aged spontaneously hypertensive rats. *Fluids and Barriers of the CNS*.

[5] Johnston M., Zakharov A., Papaiconomou C., Salmasi G., Armstrong D. (2004). Evidence of connections between cerebrospinal fluid and nasal lymphatic vessels in humans, non-human primates and other mammalian species. *Cerebrospinal Fluid Research*.

[6] Ringstad G., Vatnehol S. A., Eide P. K. (2017). Glymphatic MRI in idiopathic normal pressure hydrocephalus. *Brain*.

[7] Taoka T., Masutani Y., Kawai H. (2017). Evaluation of glymphatic system activity with the diffusion MR technique: diffusion tensor image analysis along the perivascular space (DTI-ALPS) in Alzheimer’s disease cases. *Japanese Journal of Radiology*.

[8] Jaraj D., Agerskov S., Rabiei K. (2016). Vascular factors in suspected normal pressure hydrocephalus: a population-based study. *Neurology*.

[9] Miskin N., Patel H., Franceschi A. M. (2017). Diagnosis of normal-pressure hydrocephalus: use of traditional measures in the era of volumetric mr imaging. *Radiology*.

[10] Shinohara Y., Tohgi H., Hirai S. (2007). Effect of the Ca antagonist nilvadipine on stroke occurrence or recurrence and extension of asymptomatic cerebral infarction in hypertensive patients with or without history of stroke (PICA study). *Cerebrovascular Disease*.

[11] Hattori T., Ito K., Aoki S. (2012). White matter alteration in idiopathic normal pressure hydrocephalus: tract-based spatial statistics study. *American Journal of Neuroradiology*.

[12] Hořínek D., Štěpán-Buksakowska I., Szabó N. (2016). Difference in white matter microstructure in differential diagnosis of normal pressure hydrocephalus and Alzheimer's disease. *Clinical Neurology and Neurosurgery*.

[13] Eide P. K., Sorteberg W. (2010). Diagnostic intracranial pressure monitoring and surgical management in idiopathic normal pressure hydrocephalus: a 6-year review of 214 patients. *Neurosurgery*.

[14] Greitz D. (2004). Radiological assessment of hydrocephalus: new theories and implications for therapy. *Neurosurgical Review*.

[15] Iliff J. J., Wang M., Zeppenfeld D. M. (2013). Cerebral arterial pulsation drives paravascular CSF-Interstitial fluid exchange in the murine brain. *The Journal of Neuroscience*.

[16] Yin L. K., Zheng J. J., Zhao L. (2017). Reversed aqueductal cerebrospinal fluid net flow in idiopathic normal pressure hydrocephalus. *Acta Neurologica Scandinavica*.

[17] Serulle Y., Rusinek H., Kirov I. I. (2014). Differentiating shunt-responsive normal pressure hydrocephalus from alzheimer disease and normal aging: pilot study using automated mri brain tissue segmentation. *Journal of Neurology*.

[18] Cabral J., Hugues E., Sporns O., Deco G. (2011). Role of local network oscillations in resting-state functional connectivity. *NeuroImage*.

